# The role of lipid post–translational modification in plant developmental processes

**DOI:** 10.3389/fpls.2014.00050

**Published:** 2014-02-18

**Authors:** Mark P. Running

**Affiliations:** Department of Biology, University of LouisvilleLouisville, KY, USA

**Keywords:** prenylation, farnesylation, geranylgeranylation, myristoylation, acylation, palmitoylation

## Abstract

Most eukaryotic proteins are post-translationally modified, and modification has profound effects on protein function. One key modification is the attachment of a lipid group to certain amino acids; this typically facilitates subcellular targeting (association with a membrane) and protein–protein interactions (by virtue of the large hydrophobic moiety). Most widely recognized are lipid modifications of proteins involved in developmental signaling, but proteins with structural roles are also lipid-modified. The three known types of intracellular protein lipid modifications are S-acylation, N-myristoylation, and prenylation. In plants, genetic analysis of the enzymes involved, along with molecular analysis of select target proteins, has recently shed light on the roles of lipid modification in key developmental processes, such as meristem function, flower development, polar cell elongation, cell differentiation, and hormone responses. In addition, while lipid post-translational mechanisms are generally conserved among eukaryotes, plants differ in the nature and function of target proteins, the effects of lipid modification on target proteins, and the roles of lipid modification in developmental processes.

## INTRODUCTION

With the rapid advancement of genome sequencing technologies, it is now possible to know virtually all the genes, and thus the full complement of proteins, in an increasing number of organisms. The next frontier involves learning more about these proteins: their activation/deactivation, their localization, and their function. Most proteins are post-translationally modified, and these modifications have profound implications on protein activity. This review focuses on lipid modifications of proteins. Intracellular protein lipid modifications include prenylation, N-myristoylation, and S-acylation and serve a primary role of facilitating targeting and association of the lipidated protein to various cellular membranes and subdomains of membranes, along with a secondary role in promoting protein–protein interactions between the hydrophobic chain and a hydrophobic region of an accessory protein (Thompson and Okuyama, 2000). Hundreds of proteins are known or thought to be lipid modified in plants; this review focuses on the role of protein lipid modifications in developmental signaling and plant growth processes.

## PROTEIN PRENYLATION

Of the three types of known intracellular lipid modifications of proteins in plants, protein prenylation is the most well-studied. Prenylation involves the addition of a single 15-carbon farnesyl or single or dual 20-carbon geranylgeranyl moieties to one or two cysteines near the C-terminus of target proteins (Zhang and Casey, 1996). Prenylation is conserved among Eukaryotes (Yalovsky et al., 1999; Galichet and Gruissem, 2003; Maurer-Stroh et al., 2003). Three separate heterodimeric enzymes perform prenylation: protein farnesyltransferase (PFT), protein geranylgeranyltransferase-I (PGGT), and Rab geranylgeranyltransferase (Rab-GGT, also called geranylgeranyltransferase-II; Maurer-Stroh et al., 2003; McTaggart, 2006).

Protein farnesyltransferase and protein geranylgeranyltransferase-I share a common α subunit but have distinct β subunits that are distantly related in sequence (usually 25–35% similarity) and that determine substrate specificity. PFT recognizes a C-terminal CaaX box, where C is the prenylated cysteine, a is usually aliphatic, and X is usually alanine, cysteine, glutamine, methionine, or serine. PGGT recognizes a similar sequence, except the X is almost always leucine. A wide range of critical signaling proteins is prenylated by PFT and PGGT, including members of the Ras superfamily of small GTPases, heterotrimeric G protein γ subunits, certain classes of protein kinases, and many others (McTaggart, 2006; Maurer-Stroh et al., 2007; Nguyen et al., 2010). Mutations in the Ras family are implicated in at least 15% of human cancers, and farnesyltransferase inhibitors have undergone Phase III trials as chemotherapy agents (Nguyen et al., 2010).

Rab geranylgeranyltransferase also consists of α and β subunits, which are distantly related (typically 20–30% similarity) to the PFT/PGGT α and β subunits, respectively, and also requires an additional protein, Rab escort protein (REP), for activity (Leung et al., 2006). Mammalian and yeast Rab-GGT is thought to exclusively prenylate Rab GTPases, a family of Ras-related small GTPases involved in organelle biogenesis and vesicle transport (Leung et al., 2006). Rab-GGT contain a more diverse array of C-terminal target sequences, which includes dual-modified CC, CXC, CCX, CCXX, CCXXX, and the singly modified CXXX, with C as the modified cysteine (Maurer-Stroh and Eisenhaber, 2005). Defects in Rab-GGT prenylation are also implicated in a number of human diseases (McTaggart, 2006).

Many studies over the years confirm that prenylation mechanisms are broadly conserved in plants (Galichet and Gruissem, 2003; Crowell and Huizinga, 2009; Sorek et al., 2009; **Table 1**). The β subunits and shared α subunit of PFT and PGGT are present in single copy in *Arabidopsis *(Cutler et al., 1996; Ziegelhoffer et al., 2000; Caldelari et al., 2001; Maurer-Stroh et al., 2003; Running et al., 2004; Johnson et al., 2005). Rab-GGT is less well characterized in plants, but Rab-GGT activity has been shown to be present in extracts of tomato, tobacco, and *Arabidopsis *(Yalovsky et al., 1996; Hala et al., 2005). Interestingly, the *Arabidopsis* genome contains two putative Rab-GGT α and β subunits, along with a REP homolog (Lange and Ghassemian, 2003; Maurer-Stroh et al., 2003; Hala et al., 2005, 2010; Crowell and Huizinga, 2009; **Table 1**); single copies of the α and β subunits are present in yeast and animals examined to date (Leung et al., 2006).

**Table 1:** Putative protein prenylation components in *Arabidopsis.*

Enzyme	αsubunit	αsubunitgene name(s)	βsubunit	βsubunitgene name(s)
Farnesyltransferase	PFT/PGGTα	*PLP* (At3g59380)	PFTβ	*ERA1 * (At5g40280)
Geranylgeranyltransferase	PFT/PGGTα	*PLP * (At3g59380)**	PGGTβ	*GGB * (At2g39550)
Rab geranylgeranyltransferase	Rab-GGTα	*RGTA1, RGTA2 * (At4g24490, At5g41820)	Rab-GGTβ	*RGTB1, RGTB2 * (At5g12210, At3g12070)

## PFT AND PGGT FUNCTION IN PLANTS

Genetic analysis in *Arabidopsis* has revealed important roles of PFT and PGGT in plant growth, development, and environmental responses. Mutations in the *Arabidopsis* PFT β subunit, called *enhanced* *response* to *abscisic* *acid1* (*era1*, also called *wiggum*), show increased sensitivity to abscisic acid (ABA) in the maintenance of seed dormancy and guard cell function (Cutler et al., 1996; Pei et al., 1998; Allen et al., 2002; Brady et al., 2003). Modulation of *era1* levels has been shown to preserve yield of *Brassica napus* (canola) under drought conditions in field trials (Wang et al., 2005). *era1* mutants also have several developmental defects (Running et al., 1998; Bonetta et al., 2000; Yalovsky et al., 2000a). Shoot meristems are increased in size, resulting in altered phyllotaxy, and floral meristems are wider, resulting in extra organs, particularly extra sepals and petals. In addition, *era1* mutants are slightly shorter in stature and slightly slower growing. Still, the mild phenotype of *era1* is in contrast to homologous mutants in animals, where PFT mutants are lethal (Therrien et al., 1995; Mijimolle et al., 2005), and in yeast, where PFT is required for growth (Schafer et al., 1990).

Surprisingly, mutations in the *Arabidopsis* PGGT β subunit (termed *GGB*) do not cause developmental defects and in fact are completely indistinguishable from wild type under normal growth conditions. Under exogenous ABA, *ggb* mutants show slightly enhanced responses in stomata closure, and under exogenous auxin, there is a slight enhancement of lateral root formation, but no other phenotypes are apparent (Johnson et al., 2005). This is in stark contrast to animal and yeast PGGT β subunit knockouts, which are invariably lethal (Ohya et al., 1991; Diaz et al., 1993; Trueblood et al., 1993; Therrien et al., 1995).

Mutations in the *Arabidopsis* α subunit shared between PFT and PGGT, termed *PLURIPETALA* (PLP), cause severe developmental phenotypes, including much larger shoot meristems, stem fasciation, and extra floral organs (Running et al., 2004). Flowers of *plp* mutants show about one extra organ per whorl, with the exception of petals, of which there can be up to 12 instead of the normal four found in wild type. The large meristems seen in *plp* appear to result from a defect in differentiation: primordia that are initiated from the shoot meristem occasionally fail to differentiate into a primordia fate and retain a partial or full meristem fate, initiating their own primordia and eventually fusing to the primary shoot meristem, resulting in compound shoot meristems harboring multiple central zones (Running et al., 2004). *plp* mutants also flower a month late under long days. *plp* mutants are sensitive to ABA in inhibition of seed germination, though they are not as sensitive as *era1* mutants, and also show enhanced drought tolerance (Running et al., 2004; Wang et al., 2009). Adaxial leaf pavement cells have fewer lobes in *plp *(Sorek et al., 2011). Remarkably, *plp* mutants remain viable and fertile, though with reduced seed set, while PFT/PGGT α subunit mutants are lethal in yeast (He et al., 1991) and have not been reported in non-plant eukaryotes.

It is likely that *ERA1* and *GGB* represent the only PFT/PGGT β subunits in plants, as the *era1* *ggb* double mutant phenotypically resembles *plp* (Johnson et al., 2005). The mild phenotypes of *era1* and especially *ggb* compared to *plp* suggest that there is a great deal of functional overlap in *Arabidopsis* prenylation enzymes compared to those of other eukaryotes. Cross-specificity of PFT and PGGT have been reported for a small number of target proteins in mammals and yeast, but in most cases proteins that are substrates for PFT are poor substrates for PGGT and vice versa (Zhang and Casey, 1996; Maurer-Stroh and Eisenhaber, 2005; McTaggart, 2006). Additional genetic, cytological, and biochemical evidence supports considerable cross-specificity of PFT and PGGT in plants. Overexpression of *GGB* in *era1* mutants can significantly rescue the *era1* phenotype (Johnson et al., 2005). Recombinant *Arabidopsis* PFT can prenylate several PGGT targets, including the heterotrimeric G protein γ subunits AGG1 and AGG2, several Rop-GTPases, and selected membrane-anchored ubiquitin-fold (MUB) proteins, and farnesylation is sufficient for extensive membrane localization of most of these proteins *in vivo* (Downes et al., 2006; Zeng et al., 2007; Sorek et al., 2011). *In vitro* studies show that *Arabidopsis* PFT has nearly as high affinity for leucine as it does for the standard alanine, cysteine, glutamine, methionine, and serine in the X position of the CaaX target sequence, while PGGT shows weak but measurable affinity for non-leucine terminal amino acids (Andrews et al., 2010), which helps explain both the weak *era1* and very weak *ggb* mutant phenotypes compared to *plp*. Plant prenylation enzymes appear to be less stringent in protein target sequence in general: an aromatic amino acid in the a_2_ position of the CaaX box does not hinder prenylation of the Rho-related GTPases Rac13 from cotton and ROP2 from *Arabidopsis* (Trainin et al., 1996; Johnson et al., 2005; Lane and Beese, 2006), while similar sequences act as potent inhibitors of mammalian PFT (Lane and Beese, 2006).

There are several possible explanations for the viability of *plp* mutants. One is the presence of non-prenylated proteins that can compensate for the loss of function of prenylated proteins. Another is that other lipid modifications, such as acylation (Hemsley and Grierson, 2008), may be enough to confer proper localization to a subset of prenylated proteins. Both of these may be true for ROP proteins, the Rho-related GTPases in plants (Vernoud et al., 2003). *Arabidopsis* contains 11 ROPs, 10 of which contain a CaaX motif; of these, three are either not prenylated or show only weak prenylation *in vitro* (Lavy et al., 2002; Zheng et al., 2002). Instead, these three (termed type II ROPs) are palmitoylated, and palmitoylation is necessary for their membrane localization and function (Lavy et al., 2002; Sorek et al., 2011).

## PFT AND PGGT TARGET PROTEINS

Perhaps the most surprising aspect of the viability and fertility of *plp* is the sheer number of putative PFT and PGGT protein targets that would be affected in the mutant plants. Our analysis and those of others show that over 250 *Arabidopsis* proteins fit ideal criteria for prenylation, while as many as 700 fit minimal criteria (the presense of the cysteine four residues from the C terminus; Galichet and Gruissem, 2003). Some of the functional classes of proteins found in these database searches include transcription regulation, signaling, polar cell growth, cell cycle regulation, cell wall modification, hormone synthesis and response, metal ion homeostasis, pathogen defense response, protein folding, and many of unknown function (Galichet and Gruissem, 2003; Zeng and Running, 2008; Crowell and Huizinga, 2009; Sorek et al., 2009). Some functional classes, such as prenylated transcription factors, are not found in other eukaryotes, while some proteins that are prenylated in other systems lack prenylation motifs in plants, like nuclear lamins and inosital triphosphate 5′-phosphatases (Crowell and Huizinga, 2009; Sorek et al., 2009).

An increasing number of plant proteins have been shown to be prenylated *in vitro*, and there is direct or indirect evidence for the prenylation of several proteins *in vivo* as well. Of the aforementioned ROP proteins, Class I (ROP1–ROP8) contain a classical CaaL motif and are likely prenylated *in vivo*; indeed, ROP6 has been shown to be geranylgeranylated *in vitro* and *in vivo* (Sorek et al., 2011). *Arabidopsis* ROP1, ROP2, ROP3, and ROP7 were also shown to be geranylgeranylated (Sorek et al., 2009). In the absence of PGGT, ROP6 can also be farnesylated *in vitro* and *in vivo*. ROP proteins from other species have also been shown to be prenylated, including ROP1Ps from pea and Rac13 from cotton (Trainin et al., 1996).

The *Arabidopsis* heterotrimeric G protein γ subunits AGG1 and AGG2 have been shown to be prenylated (Adjobo-Hermans et al., 2006; Zeng et al., 2007). Like ROP6, both AGG1 and AGG2 contain a CaaL motif for geranylgeranylation, but they can also be farnesylated *in vitro*. Their membrane localization is only slightly affected in a *ggb* mutant, indicating again a compensation for the loss of PGGT by PFT, but is drastically altered in a *plp* mutant (Zeng et al., 2007). Interestingly, a third, atypical heterotrimeric G protein γ subunit, AGG3, was recently identified, but, despite its plasma membrane localization, it lacks a prenylation motif and is likely not prenylated (Chakravorty et al., 2011).

There are six MUB protein family members in *Arabidopsis*, five of which have a CaaX or CaaL box and can be prenylated *in vitro* (Downes et al., 2006). All six *Arabidopsis* MUBs are associated with the plasma membrane *in vivo*. One difference observed with MUB6, which has a CaaL box, is that its plasma membrane localization is lost in a *ggb* mutant, suggesting that PFT cannot compensate for the loss of PGGT for all target proteins.

The *Arabidopsis* nucleosome assembly protein AtNAP1;1 has been shown to be farnesylated *in vivo* (Galichet and Gruissem, 2006). AtNAP1;1 also provides the only known example for which farnesylation is developmentally regulated in plants, and non-farnesylated AtNAP1;1 promotes cell expansion versus cell division in later stages of leaf development (Galichet and Gruissem, 2006).

Plants also harbor the only known prenylated transcription factors, including APETALA1 protein family members (Yalovsky et al., 2000b). *Arabidopsis* *apetala1* mutant flowers show a lack of petals and a conversion of sepals to leaf-like bracts, with production of additional flowers within the flower (Mandel et al., 1992). This is different than the *plp* floral mutant phenotype, making it unclear what the role of prenylation is for APETALA1 function. However, prenylation of APETALA1 is required for the overexpression phenotype of a conversion of apical meristem to floral meristem fate (Yalovsky et al., 2000b).

A variety of other plant proteins have been shown to be prenylated *in vitro*, including the *Arabidopsis* metal binding domain proteins ATFP3 and HIPP26, the tobacco disease resistance-related protein NTGP4, tobacco and *Arabidopsis* homologs of the yeast v-SNARE protein Ykt6p, petunia calmodulin protein CaM53, the *Arabidopsis* AUX/IAA family member IAA4, and *Arabidopsis* AtIPT3 (Barth et al., 2009; Crowell and Huizinga, 2009). Disappointingly, however, there is no indication that any of the currently known prenylated proteins play any role in the striking developmental phenotype of *plp* mutants, suggesting that as-yet-unidentified target proteins play specific roles in meristem function and flower development.

## Rab-GGT FUNCTION IN PLANTS

While PFT and PGGT mutants have been available for many years, only recently have mutants in one of the Rab-GGT subunits been characterized. Mutations in the Rab-GGT beta subunit RGTB1 gene result in a striking series of phenotypes, including smaller, epinastic leaves, loss of apical dominance (extreme branching), greatly delayed senescence, and infertility, though both male and female gametes can be effectively used in crosses (Hala et al., 2010). The mutant results in the accumulation of the unprenylated form of at least one Rab protein, Rab A2a. The mutants also show shoot gravitropic defects and a constitutive photomorphogenic phenotype in the dark, but surprisingly, little or no effects on root growth (Hala et al., 2010).

The effects of mutations in the other beta subunit, RGTB2, and in either of the alpha subunits RGTA1 and RGTA2, have not been reported. It would be interesting to see, for instance, if the *rgtb1,* *rgtb2* double mutant is lethal. RGTB2 mRNA is present but is one-tenth as abundant as RGTB1 mRNA (Hala et al., 2010), suggesting that residual Rab-GGT activity may be responsible for the survivability of *rgtb1* mutants. Another intriguing possibility is if some Rab proteins with a CaaX motif can be efficiently prenylated by PFT or PGGT, compensating for the loss of Rab-GGT function. There is some precedence for this: mammalian Rab8, with a CVLL motif can be efficiently prenylated by PGGT *in vitro* (Wilson et al., 1998), and Rab11 (CQNI) can be prenylated by PFT (Joberty et al., 1993). Indeed, 32 of the 56 *Arabidopsis* Rab proteins contain a CXXX motif that can in theory act as a PFT target. Interestingly, at least one Rab protein, ARA6, is not prenylated in *Arabidopsis*; instead, it is myristoylated and palmitoylated (Ueda et al., 2001) and is likely to function in the absence of Rab-GGT activity. While RGTA1 is expressed everywhere, RGTA2 is expressed only in the pollen (Hala et al., 2010), suggesting that, if indeed Rab-GGT is required for survival, viable *rgta1* complete loss of function mutants may be more difficult to isolate.

## N-MYRISTOYLATION

N-myristoylation involves the addition of a 14 carbon saturated myristate group to the N-terminal glycine of target proteins (Gordon et al., 1991; Johnson et al., 1994). It is carried out by a monomeric enzyme, *N*-myristoyltransferase (NMT; Duronio et al., 1989). The absolute requirement for enzymatic activity is an N-terminal glycine on the target protein, while amino acid residues two through six are used in substrate recognition, residues 7 through 10 are less restricted, and residues 11 through 17 are hydrophilic (Maurer-Stroh et al., 2002; Utsumi et al., 2004). Interestingly, experimental evidence as well as homology remodeling of NMT protein structures suggest that there are substrate specificity differences among different species (Maurer-Stroh et al., 2002).

*Arabidopsis* contains two homologs of NMT, termed *AtNMT1* (At5g57020) and *AtNMT2* At2g44170 (Qi et al., 2000), and AtNMT1 protein has been shown to have myristoylation activity on N-terminal glycine-containing peptides (Qi et al., 2000; Boisson et al., 2003; Podell and Gribskov, 2004). In fact such studies have allowed a refinement of the predicted *Arabidopsis* myristoylation target sequence, which is somewhat distinct from that of animals and fungi (Boisson et al., 2003; Podell and Gribskov, 2004).

Several lines of evidence indicate that AtNMT1 is the primary N-myristoylation enzyme in *Arabidopsis*. Its mRNA is expressed at a higher level than that of *AtNMT2* (Pierre et al., 2007), and indeed it remains unclear if AtNMT2 has any myristoylation activity, despite showing 80% identity to AtNMT1 (Boisson et al., 2003). *AtNMT1* knockouts are lethal at an early stage (Pierre et al., 2007), suggesting that AtNMT2 cannot compensate for loss of AtNMT1 activity in *Arabidopsis*. In addition, constitutive expression of AtNMT2 in an *AtNMT1* mutant also fails to rescue (Pierre et al., 2007), indicating that *AtNMT2* truly has a different function than *AtNMT1*. Unlike *AtNMT1*, *AtNMT2* cannot complement a yeast *nmt1* mutant, and AtNMT2 cannot myristoylate at least one AtNMT1 target, SALT OVERLY SENSITIVE3 (Boisson et al., 2003).

## AtNMT1 AND AtNMT2 FUNCTION IN PLANTS

A knockout of the *AtNMT1* gene results in a seedling lethal phenotype: seedling arrest occurred at day 4 of germination (Pierre et al., 2007). In addition, seedlings were hypersensitive to high glucose concentration. Interestingly, the roots and root meristem were normal in this mutant, but the rib zone of the shoot meristem was completely disorganized, suggesting a role in shoot meristem establishment for *AtNMT1* (Pierre et al., 2007). Lack of AtNMT1 expression at later time points shows that AtNMT1 is required for most aspects of post-embryonic development as well, including flower differentiation, fruit maturation, and fertility (Pierre et al., 2007). A weak allele of *AtNMT1* shows defects in Golgi trafficking and integrity (Renna et al., 2013). The loss of *AtNMT2* was much less severe than the loss of *AtNMT1* and resulted strictly in a delay of flowering (Pierre et al., 2007). However, expression of *AtNMT2* under the *AtNMT1* promoter resulted in shoot meristem defects.

## MYRISTOYLATION TARGETS IN PLANTS

As is the case for prenylation, proteins shown or predicted to be myristoylated by NMT1 *Arabidopsis* include several protein classes that are also myristoylated in other eukaryotes, as well as novel classes (Boisson et al., 2003; Podell and Gribskov, 2004; Martinez et al., 2008). Of the 319 Arabidopsis proteins predicted to be myristoylated by Podell and Gribskov (2004), 77 are kinases (including most of the known calcium-dependent protein kinases), 13 are phosphatases, 15 are GTP-binding proteins, 10 are transcription factors, and 132 are unknown proteins.

In recent years the number of plant proteins shown experimentally to be N-myristoylated, either directly (by *in vitro* myristoylation assays) or indirectly (by mislocalization of the protein when the N-terminal glycine is mutated) has increased significantly (**Table 2**). The developmental arrest of *Atnmt1* knockouts appears to be due primarily to the loss of myristoylation on SnRK1 kinase (Pierre et al., 2007), but in other cases it is unclear how these myristoylated proteins contribute to the *Atnmt1* phenotype.

**Table 2:** Myristoylated proteins.

Protein	Species	Function	Reference
SnRK1	*Arabidopsis*	Protein kinase	Pierre et al. (2007)
ARA6	*Arabidopsis*	RabGTPase	Ueda et al. (2001), Boisson et al. (2003)
SOS3	*Arabidopsis*	Calcinurin B-like protein	Ishitani et al. (2000)
PTO	Tomato	Protein kinase	De Vries et al. (2006)
CBL1	*Arabidopsis*	Calcinurin B-like protein	Batistic et al. (2008)
AtPCaP1	*Arabidopsis*	Cation-binding protein	Nagasaki et al. (2008)
NtCDPK2	Tobacco	Calcium-dependent protein kinase	Witte et al. (2010)
CPK3	*Arabidopsis*	Calcium-dependent protein kinase	Mehlmer et al. (2010)
AtTrx*h*9	*Arabidopsis*	h-type thioredoxin	Meng et al. (2010), Traverso et al. (2013)
BON1	*Arabidopsis*	Copine protein	Li et al. (2010)
CBL4	*Arabidopsis*	Calcium sensor	Held et al. (2011)
CAST AWAY	*Arabidopsis*	Cytoplasmic receptor kinase	Burr et al. (2011)
RPP1-WsB	*Arabidopsis*	Leucine-rich repeat	Takemoto et al. (2012)
RPS5	*Arabidopsis*	Nucleotide-binding	Takemoto et al. (2012)
PBS1	*Arabidopsis*	Protein kinase	Takemoto et al. (2012)
POLTERGEIST	*Arabidopsis*	2C-type protein phosphatase	Gagne and Clark (2010)
POLTERGEIST-LIKE	*Arabidopsis*	2C-type protein phosphatase	Gagne and Clark (2010)
PP2C74	*Arabidopsis*	2C-type protein phosphatase	Tsugama et al. (2012b)
PP2C52	*Arabidopsis*	2C-type protein phosphatase	Tsugama et al. (2012a)
PCaP1	*Arabidopsis*	Cation binding protein	Vijayapalani et al. (2012)
CAN1	*Arabidopsis*	Nuclease	Lesniewicz et al. (2012)
CAN2	*Arabidopsis*	Nuclease	Lesniewicz et al. (2012)
GPA1	*Arabidopsis*	Heterotrimeric G protein alpha subunit	Boisson et al. (2003), Feng et al. (2013)

## PROTEIN S-ACYLATION

Protein S-acylation is the covalent attachment of a fatty acid to a cysteine residue. Commonly the acyl group involved is palmitate, a 16-carbon saturated fatty acid, but other groups, including shorter or longer or unsaturated groups, can be added (Hallak et al., 1994). Unlike prenylation and N-myristoylation, S-acylation is reversible (Resh, 2006). Also unlike prenylation or myristoylation, which have specific target sequences at the C- and N-terminus of the protein, the prenylated cysteine can be localized anywhere in the protein, though it is common to find acylated cysteines in close proximity to myristoylated glycines on the N-terminus or prenylated cysteins on the C-terminus (Resh, 2006).

In contrast to prenylation or N-myristoylation, a large protein family is responsible for S-acylation. *Arabidopsis* harbors 24 putative protein S-acyltransferases (PATs), which are characterized by having four to six transmembrane domains and a DHHC amino acid motif within a cysteine rich domain (Hemsley and Grierson, 2008; Hemsley, 2009; Batistic, 2012). Several of these 24 have been confirmed to have acyltransferase activity (Batistic, 2012). Interestingly, the 24 PATs are localized to a variety of different intracellular membranes, although the majority is localized at the plasma membrane (Batistic, 2012).

## PAT FUNCTION IN PLANTS

Despite the high number of PATs in *Arabidopsis*, they do not act completely redundantly; two of the PATs have been reported so far to have visible phenotypes when mutated. The first mutation in a PAT, called *tip1*, was originally identified in a screen for altered root hair phenotypes (Schiefelbein et al., 1993; Ryan et al., 1998). The root hairs of *tip1* mutants are wider, shorter and often branched at their base. Pollen, another cell type that undergoes polar elongation, is affected in both germination and growth, resulting in reduced male fertility (Schiefelbein et al., 1993; Ryan et al., 1998). *tip1* mutants also show general growth defects, including shorter stature and smaller rosettes (Ryan et al., 1998). Interestingly, *TIP1* (At5g20350) encodes the only PAT in *Arabidopsis* that also contains ankryin repeats, a total of six at the N-terminus; ankryin repeats play a role in protein–protein interactions (Hemsley et al., 2005).

Mutations in a second PAT, *PAT10* (At3g51390), has recently been described. The PAT10 protein is localized to the tonoplast and possibly the Golgi and trans-Golgi network, and mutations in *PAT10* result in pleiotropic effects on growth and environmental response (Qi et al., 2013; Zhou et al., 2013). *pat10* mutants are smaller and grow much more slowly than wild type, with much smaller rosette leaves and inflorescence stems, a consequence of both reduced cell division and cell expansion (Qi et al., 2013; Zhou et al., 2013). Vascular development is also affected (Qi et al., 2013). Fertility is much reduced, a consequence of short stamens, pollen coat defects, pollen tube elongation and targeting defects, and disorganized embryo sacs (Zhou et al., 2013). *pat10* mutants are also salt sensitive (Zhou et al., 2013).

## S-ACYLATION TARGETS IN PLANTS

Identification of S-acylated proteins in plants using bioinformatics is not feasible due to the lack of a consensus sequence for acylation. However, a recent study using a combined proteomics/bioinformatics approach identified about 600 proteins putatively palmitoylated, much more than previously thought (Hemsley et al., 2013). These include a proteins with a diverse array of biochemical functions, including MAP kinases, leucine-rich repeat receptor kinases, integral membrane transporters, ATPases, and SNAREs, many of which were hitherto not suspected of being acylated. This study also confirmed that S-acylation is important for the function of FLS2, a leucine-rich repeat receptor like kinase involved in pathogen perception (Hemsley et al., 2013).

Previous studies have focused on the role of S-acylation of particular target proteins. In some cases S-acylation compensates for the lack of prenylation in targeting proteins to the membranes. The three type II ROPs of *Arabidopsis*, AtROP9, AtROP10, and AtROP11 are S-acylated in lieu of prenylation, and S-acylation is required for their correct membrane localization (Lavy et al., 2002). AtMUB2 also is likely S-acylated via its cysteine-rich C-terminus and is targeted to the membrane (Downes et al., 2006).

More commonly, though, S-acylation occurs in conjunction with either prenylation or myristoylation. In some examples, either prenylation or myristoylation is sufficient to target proteins to an internal membrane, while further S-acylation is required to confer plasma membrane localization. For instance, in the case of AGG2, disruption of a putative S-acylated cysteine site two amino acids upstream of the prenylated cysteine causes localization to the trans-Golgi, while full plasma membrane localization is restored when both cysteines are intact (Zeng et al., 2007). Interestingly, disruption of the putatively acylated cysteine in the same position in AGG1 does not cause a change in membrane localization (Zeng et al., 2007). Similarly, in the case of h-type thioredoxin, myristoylation alone confers localization to the ER/Golgi, and S-acylation is required for localization to the plasma membrane (Traverso et al., 2013).

Several other proteins previously mentioned are dual modified, including ARA6 (Ueda et al., 2001), type I ROPs (Sorek et al., 2007, 2011), CBL1 (Batistic et al., 2008), NtCDPK2 (Witte et al., 2010), POLTERGEIST and POLTERGEIST-LIKE (Gagne and Clark, 2010), CBL4 (Held et al., 2011), RPS5 (Qi et al., 2012), and CAN1 and CAN2 (Lesniewicz et al., 2012).

Since PAT10 and TIP1 act non-redundantly, and the other PATs cannot completely compensate for their function, it follows that at least some of their respective target proteins are unique. In the case of PAT10, it was noted that certain *CBL1*-related genes also have putative S-acylation sites, are also localized to the tonoplast and, like *PAT10*, are involved in abiotic stress responses (Zhou et al., 2013). Indeed, CBL2, CBL3, and CBL6 are all cytoplasmically localized instead of tonoplast-localized both in *pat10* mutants and when wild type plants are treated with the palmitoylation inhibitor 2-bromopalmitate, making these excellent candidates for PAT10 targets (Zhou et al., 2013). In the case of TIP1, it is possible to compare the relative S-acylation levels of a protein between wild type and *tip1* mutant samples. One hundred three proteins showed a greater than or equal to 1.5 fold decrease in S-acylation in the mutant, indicating that they are either targets of TIP1 or they may show reduced acylation indirectly as a consequence of the developmental effects of *tip1* mutants (Hemsley et al., 2013).

## CONCLUSIONS AND FUTURE PROSPECTS

Much progress has been made in understanding the processes of protein lipidation in *Arabidopsis* in the past decade. In the case of prenylation and N-myristoylation, *Arabidopsis* target sequences have been refined, which is important as they vary somewhat from non-plant eukaryotes, and a large scale proteomics effort have uncovered a wealth of S-acylation targets, though target sequences for S-acylation remains elusive. We now have in hand *Arabidopsis* mutations for many of the key lipidation enzymes, though it would be a welcome advance to differentiate a role for putative Rab-GGTase subunits RGTB2, RGTBA1, and RGTBA2, and it will be interesting to see if other PATs have phenotypes other than the two reported. It is likely that further information will come from double mutant studies; for instance, whether *rgtb1* *rgtb2* double mutants are lethal, and to uncover putative redundancies within the PAT family. Now that information for expression and subcellular localization of PATs are at hand (Batistic, 2012), it may be easier to identify possible redundant factors.

A wealth of information is now available for lipidated proteins; however, those that have been confirmed via *in vitro* and *in vivo* assays and subcellular localization studies still represent only a small percentage of proteins that are likely to be lipidated. More high-throughput studies on identification of prenylated or N-myristoylated proteins would be helpful. Further characterization of additional target proteins will help clarify the multiple roles of lipidation in subcellular localization and protein function.

One gaping hole in our knowledge on plant protein lipidation is that almost all the studies of knockout and knockdown mutants in lipidation enzymes in plants has been in *Arabidopsis*. It is not known, for instance, if the lack of both farnesylation and geranylgeranylation (represented by *plp* mutants) is more generally lethal in plants (as it is in other eukaryotes), and *Arabidopsis* represents an outlier in its function. Similarly with the other prenylation, N-myristoylation, or S-acylation enzymes, it is not known whether their biological function is conserved among plants. Knockouts or knockdowns of lipidation enzymes in species other than *Arabidopsis* will help answer these questions.

## Conflict of Interest Statement

The author declares that the research was conducted in the absence of any commercial or financial relationships that could be construed as a potential conflict of interest.
